# Impact of Ethical Leadership on Autonomy and Self-Efficacy in Virtual Work Environments: The Disintegrating Effect of an Egoistic Climate

**DOI:** 10.3390/bs15010095

**Published:** 2025-01-20

**Authors:** Carlos Santiago-Torner, José-Antonio Corral-Marfil, Yirsa Jiménez-Pérez, Elisenda Tarrats-Pons

**Affiliations:** 1 Department of Economics and Business, Faculty of Business and Communication Studies, University of Vic—Central University of Catalonia, 08500 Vic, Spain; elisenda.tarrats@uvic.cat; 2 Department of Psychology, Faculty of Education, Translation, Sport and Psychology, University of Vic—Central University of Catalonia, 08500 Vic, Spain; yirsa.jimenez@uvic.cat; 3 Department of Social Psychology and Quantitative Psychology, Faculty of Psychology, University of Barcelona, 08007 Barcelona, Spain

**Keywords:** ethical leadership, job autonomy, self-efficacy, ethical climate, egoistic climate

## Abstract

Ethical management is key to ensuring organizational sustainability, through resources such as autonomy or self-efficacy. However, economic and social uncertainty occasionally leads to adaptive responses that prioritize profit as the primary interest, blurring the integrating role of ethical leadership. There are a number of studies that support this reality in a virtual work environment. This sector-specific and cross-sectional research explores how ethical leadership influences self-efficacy among teleworkers, through active commitment to job autonomy, and how an egoistic climate hinders this influence. The analysis is quantitative and correlational, and the sample includes 448 teleworkers. A model of conditional indirect effects, including both a mediation process and a moderation process, is used. The results support that ethical leadership enhances followers’ self-efficacy through a redistribution of responsibilities, which increases the perception of autonomy. However, when ethical leadership coincides with a climate that has opposing interests, such as an egoistic climate, ethical leadership is unable to counteract it, and its effect on self-efficacy gradually diminishes. The benefits of this management style are widely known, but it is crucial to understand under what circumstances it loses efficacy. This research presents a new theoretical model that contributes to the existing literature on ethical leadership. Lastly, organizations that embrace ethical leadership can avoid the emergence of ethical climates disconnected from collective benefit, such as those characterized by selfishness, which hinder prosocial motivation. In this context, ethical leadership fosters the development of high-quality interpersonal relationships with followers, which are considered essential for creating an environment conducive to group learning. Consequently, change management in organizations necessitates the adoption of an ethical system that enhances self-efficacy through moral principles, rather than relying solely on individualistic aspects.

## 1. Introduction

Colombia is immersed in a political and economic context of instability that has become the main strategic challenge for most organizations. In this sense, ethical leadership is a key factor that tends to balance certain external risks while conserving resources and building new development sources (Markey et al., 2021). Colombian industry needs a leadership style capable of articulating the changes in the way most companies operate that resulted from COVID-19. In fact, telework has been incorporated as a necessary adaptative system and this modality of occupation requires a type of behavior supported by strong moral convictions (Santiago-Torner, 2023a). Therefore, ethical leadership becomes a valuable management style that avoids passive behaviors under hostile or unstable habitats or with limited resources (Allal-Chérif et al., 2021).

Dhar (2016) clarifies that ethical leadership focuses part of its efforts on defining exchange relations with employees, and this interaction alters characteristics related to the job position. Job autonomy specifically plays a critical role in building trust and contributing to increased individual self-efficacy (Paredes-Aguirre et al., 2024; Wattoo et al., 2020). Autonomy and self-efficacy are interconnected with resilience, and with the individual’s ability to regulate and respond with adaptative solutions to a crisis environment (Salanova et al., 2022). Therefore, both skills have a useful impact on stress management and a positive impact on psychological well-being, allowing employees to face demanding situations with guarantees of success (Zito et al., 2021). On the other hand, prolonged periods of imbalance can lead to organic responses that benefit the performance and self-efficacy of organizational structures to the detriment of ethical management (Tanner et al., 2015). Surely, self-interest and institutional interest, when permanent, shape egoistic ethical climates that primarily rely on competitive states of mind (Brändle et al., 2018). Consequently, benefit is the main concern, which differs from the integrating criteria of ethical leaders (Al Halbusi et al., 2021).

This research was started under a complex scenario with multiple questions. The first is how to define the relationship between ethical leadership and self-efficacy, considering the critical contextual aspect that this possible affinity is established in virtual work environments. Digital technologies, including social media, artificial intelligence, and cloud data storage, have significantly transformed workplace dynamics, particularly the behaviors of leaders in virtual work environments. E-leadership is defined as a process of social influence, exercised in both proximal and distal settings, that can transform workers’ attitudes, emotions, thoughts, behaviors, and performance (Avolio et al., 2014).

The literature on human resource management highlights that a virtual work environment creates a complex interaction between digital technologies, leadership, and specific ethical behaviors of employees (Roman et al., 2019). Indeed, the digitalization of organizational processes necessitates a radical transformation of organizational culture, directly impacting communication between leaders and subordinates. This is because a virtual work environment requires a leadership style that incorporates a critical understanding of the ethical challenges and dilemmas associated with artificial intelligence, self-organization, and low interdependence (Havens, 2018). In this context, electronic ethics, or e-ethics, is associated with leaders who, in addition to promoting ethical virtues by example, are capable of adapting and reinventing themselves without compromising the organization’s ethical principles (Riivari & Lämsä, 2019). Ethics, beyond being a matter of beliefs, must align with a concrete work design where ethical leaders can play a decisive role through simple yet effective actions (Muhamamd et al., 2020). Therefore, the primary aim of ethical leadership is to provide a solution to the erosion of moral authority that characterizes virtual work environments.

According to Vaja (2017), ethical leaders have a responsibility to establish technological management frameworks that prioritize ethics, promote the conscious use of technology, and ensure that technological decisions align with the values and norms of the organization as well as the broader community. These leaders play a key role in fostering a positive technological environment by providing ethical guidance and oversight. In this context, e-ethics and the development of moral conduct in a virtual work environment largely depend on the actions of leaders. Leaders can contribute to a culture of ethical behavior in virtual settings by fostering an environment that encourages transparency and supports ethical communication (Hosseini & Ferreira, 2023). Finally, leaders can motivate employees to adopt ethical norms and values by modeling ethical behavior and cultivating an atmosphere of trust that prioritizes integrity in the workplace (Jayavelu et al., 2024).

It is important to note that the term e-ethics was developed by Lee (2009) to describe ethical leadership adapted to remote work settings. The term e-ethics is part of a broader concept of digital leadership known as e-leadership, which involves the development of specific managerial skills to optimize the management of remote work environments (Roman et al., 2019). Therefore, it is of great interest to understand how ethical leadership creates working conditions that keep employees motivated and enhance their self-efficacy, rather than diminishing it. At the same time, another particularly important aspect this research aims to address is how an egoistic ethical climate, acting as a moderating construct, influences the relationship between ethical leadership and self-efficacy. An egoistic ethical climate emerges in organizations that promote rules with a clear focus on individual benefits. In other words, in organizations characterized by a selfish orientation, self-interest is central to determining what constitutes ethically appropriate behavior. Selfish norms signal to employees that factors enhancing profitability and personal interest should take precedence over concerns about the impact on others. For this reason, an egoistic ethical climate can lead employees to cognitively distort their behaviors, through a strong moral detachment, to justify their actions (Overall & Gedeon, 2023). In fact, when an organization supports selfish behaviors and attitudes, it creates an environment where individuals may feel justified in lying, deceiving, or stealing, as the primary moral reasoning becomes disconnected from the group interest (Gorsira et al., 2018a).

An egoistic ethical climate sends contextual signals that influence employees’ moral behavior. In this regard, the ethical infrastructure of the organization, along with its reward and control systems, signals to employees what is valued and expected of them. In other words, an egoistic ethical climate becomes an organizational pressure mechanism that influences how employees assess their ethical behaviors, prioritizing effectiveness (Bhattacharyya et al., 2021). Actions aimed at promoting organizational and individual effectiveness may violate moral principles, laws, or established conduct norms. In this context, the obsession with projecting a positive organizational image is a behavior that often leads to the distortion of the truth, which is clearly aligned with an egoistic ethical climate (Graham et al., 2020).

Authors such as Tziner et al. (2015) conclude that an egoistic ethical climate is not significantly related to the theory of social exchange between leader and follower (LMX). This indicates that a managerial pattern that originates in quality interactions, mutual influence, and respect ceases to be effective when the moral balance focuses exclusively on self-interest or organizational interest. Additionally, the same authors find that an egoistic climate is negatively related to perception of organizational justice. Likewise, Gorsira et al. (2018b) establish that employees who feel immersed in an egoistic ethical climate have weaker relationships with personal and social norms. This can subordinate their moral conception to dishonest behaviors. Considering that ethical leaders justify their functional character through social relations, justice, and integrity, it is possible to conclude that an egoistic climate continuously hinders and limits the scope of ethical leadership on job self-efficacy until this relationship is interrupted (Al Halbusi et al., 2021).

However, ethics as a moral measure does not prevent a climate with egoistic motivations from being self-effective (Tanner et al., 2015). The underlying moral currents are unquestionable in most organizations, although there is ambiguity when it comes to defining an approach as ethical or not (De Cremer et al., 2011). Therefore, traits such as responsibility or level of energy devoted to achieving an objective, through positive and useful self-efficacy, are conditioned and depend on organizational behavior and how it faces the uncertainty related to the business. An egotistic climate, for practical purposes, designs a work framework that implies strong rivalry to achieve goals. This fosters a persistent will, ethical or not, to leave behind any difficulties interfering with individual self-efficacy (Vardaman et al., 2014). Therefore, this research considers that an egoistic ethical climate significantly articulates with personal self-efficacy.

Another question this article intends to answer is how job autonomy intervenes to facilitate the correspondence between ethical leadership and self-efficacy in the job position. Considering this, self-determination, or a feeling of autonomy to complete specific tasks fits the definition of psychological empowerment (Frazier & Jacezko, 2021). After all, individual conviction of self-efficacy significantly influences organizational results. Thus, the ethical leader’s behavior is decisive, since they become role models giving meaning to the social context. Meaning that the leader provides relevant moral information from two different perspectives: personal and managerial (O’Keefe et al., 2019). Therefore, if followers modify their convictions and make them coincide with organizational values, they are more likely to find meaning in their work and become more self-efficient.

Ethical leaders naturally foster analytical and independent work environments where followers take charge of their own decisions. This control over tasks enables employees to be generally autonomous (Dust et al., 2018). Finally, and by integrating concepts, ethical leaders have a real interest in caring for the well-being of followers. Consequently, interaction between them and employees is constant, which enables a spiral of questions and answers resulting in more individual and collective self-efficacy (Bedi et al., 2016; Liu et al., 2020). This research focuses on the Colombian electricity sector, which is a key part of the sustained development of a territory that had not stopped growing until now.

The economic activity examined has certain peculiarities and standing out among them is the high academic level of its members. In fact, the entire analysis sample is formed by people with university studies and the vast majority are employees who essentially rely on information and communication technologies. Job autonomy is a main component of this perspective, as professional employees have more internal influence potential than other groups of employees (Väänänen et al., 2020). Additionally, work environment flexibility is a central factor of occupational well-being and tends to guarantee greater self-efficacy (Dedahanov et al., 2019). Likewise, leadership in this sector constantly faces ethical problems. Therefore, it requires a moral vision as it directly affects people and the rest of the organization (Páez & Salgado, 2016). To the same extent, along with ethical leadership, there are variables that have the capacity to reduce its impact (R. Khan et al., 2016). In this sense, an egoistic ethical climate stands out as it relates to corruption (Gorsira et al., 2018b), job dissatisfaction (Gencoglu & Dinc, 2017) and low organizational commitment (Saygili et al., 2020).

In conclusion, this article intends to examine how ethical leadership, exercising its function in a virtual work environment, transcends in the self-efficacy of the follower. To give more meaning to this relationship, a scheme is used that includes a mediating factor, generally positive, as job autonomy, and another potentially adverse factor, such as an egoistic ethical climate with a moderating function. This research intends to contribute, with original results, to the existing literature in different ways, overcoming several limitations evident within this field of study: first, by using ethical leadership as a construct that improves employee self-efficacy in virtual contexts; and second, by revising how and in what situations this relationship occurs or stops.

In this regard, the relationship between ethical leadership and self-efficacy has been widely analyzed (Aftab et al., 2023; Ilyas et al., 2020; Ren & Chadee, 2017). Various studies conclude that ethical leaders can guide employees’ positive attitudes towards their daily tasks, thereby enhancing their effectiveness. However, to the best of our knowledge, none of these studies have been conducted in a virtual work environment. On the other hand, the relationship between ethical leadership and self-efficacy has been established through the mediating effect of leader-member exchange, organizational trust, and commitment (Walumbwa et al., 2011; Wibawa & Takahashi, 2021). Nevertheless, to date, job autonomy has not been used to justify this relationship. Finally, after an extensive search, we have not found any studies linking ethical leadership and self-efficacy through the moderating effect of an egoistic ethical climate. In this regard, our study provides solid empirical evidence supporting the detrimental role of an egoistic ethical climate, which may offer significant practical implications and fill an important knowledge gap.

### Contextualization of the Study

The Colombian electricity sector is crucial as a driver of the country’s development, as it contributes to economic growth, generates employment, and stimulates the economy. In fact, electricity is fundamental to the lifestyle of modern society, as it not only provides lighting but is also essential for heating, cooling, and maintaining critical medical systems. In this regard, Colombia, and specifically its energy sector, is a global benchmark, as the country ranks 25th among 115 nations with the greatest progress in energy transition, according to the World Economic Forum’s Energy Transition Index. In Colombia, 83.4% of the energy generated comes from renewable sources, while 16.6% is non-renewable.

On the other hand, the Colombian electricity sector has been involved in numerous political and institutional corruption scandals. In this context, the Collective Action for Ethics and Transparency was established in 2015, with the primary goal of combating corruption through the implementation of best practices within a stringent regulatory framework. Furthermore, the new vision for the Colombian electricity sector is based on an ethical leadership style, with the main objective of properly managing the regulatory framework to enhance employee efficiency without compromising their well-being (Santiago-Torner et al., 2024c). Consequently, the Colombian electricity sector is particularly significant in the country and stands out markedly from other industries that have a lesser impact on the economy and society.

In 2008, Colombia became a pioneer in Latin America with Law 1221, which recognized teleworking as a legitimate work modality. Paradoxically, the Colombian electricity sector began implementing teleworking in mid-2020. In fact, the new leadership in the sector, considering its historical context, was hesitant to adopt a work modality that is difficult to manage and relies on trust-based relationships (Segbenya & Okorley, 2022). Therefore, it is particularly important to analyze whether virtual working conditions positively impact the growth of the Colombian electricity sector. The conclusions drawn from this new work modality could be useful not only for Colombia but also for other sectors in Latin America that are or have been involved in situations of low transparency, such as bribery, low efficiency, or unethical practices associated with corruption.

## 2. Theoretical Framework

### 2.1. Ethical Leadership, Autonomy and Self-Efficacy

There is growing interest in investigating the relationship between ethical leadership and desirable follower behaviors, such as self-efficacy, creativity, or innovation (Gabler & Kalra, 2024; K. Khan et al., 2021). However, Brown et al. (2005) and, in particular, Brown and Treviño (2006), in a review addressing the future directions of ethical leadership, suggest that it is necessary to examine more closely the mediating or moderating mechanisms that influence these relationships. In this regard, this article provides a significant novelty, as the mediating effect of job autonomy has hardly been analyzed. For example, Liu et al. (2020) use job autonomy to explain the relationship between ethical leadership and creative deviance. However, job autonomy has usually been used as a circumstance (moderation) and not as a process or mechanism (mediation) capable of clarifying a specific association, such as the effect of ethical leadership on self-efficacy.

Kalshoven et al. (2013) argue that ethical leaders are especially inclined towards work models with a high context of autonomy. In light of this, Contreras et al. (2020) indicate that job autonomy enables a free choice of tasks, which directly influences upon an adequate administration of time and improves individual self-efficacy. However, e-leaders have the responsibility of proposing transparent digital disconnection policies to prevent intensifying workloads under the false pretext of labor flexibility (Gálvez et al., 2020). Therefore, ethical leaders are necessary because, in an environment based on self-management, they guide and help followers decide what is most convenient for their well-being (Janfada, 2017). Similarly, the impact of ethical leaders depends on their ability to transfer sufficient authority to employees and to emphasize two basic aspects with this action: shared trust and explicit development of the role of followers (Bai et al., 2019).

On the other hand, considering the approach of the social learning theory (Bandura, 1986; Bandura et al., 2001), job autonomy provides employees with the ability to analyze the effect of their actions, develop points of view, and learn based on experience. Consequently, high job autonomy provides individuals with greater guidance and confidence, which allows them to understand the evolution of their own actions and how these lead them to more self-efficacy (Dedahanov et al., 2019). In fact, employees perceive a decrease in their ability to produce positive results when they experience excessive supervision that limits their potential to be able to decide (Bakker et al., 2011).

Thus, by adopting the role of e-leader ethical leadership promotes collaborative work, which feeds on multiple shades and observations, has a significant impact on job autonomy, and, using information and communication technologies, it is very possible for the follower’s self-efficacy to improve (Sudiana & Saputra, 2021). Therefore, the following hypothesis is proposed:

**H1.**  *Job autonomy, in virtual work environments, is a valuable mechanism that explains how ethical leadership and self-efficacy relate.*

### 2.2. Ethical Leadership and Job Self-Efficacy

Surprisingly, the impact of leadership on self-efficacy in a virtual work environment has gone completely unnoticed by the scientific community. For example, Alkhayyal and Bajaba (2023) establish a relationship between e-leadership, job well-being, and job performance using self-efficacy as a mediating mechanism. However, this research utilises some general competencies of electronic leadership but not the specificities of a particular leadership style such as ethical leadership. Similarly, Purnomo et al. (2023) analyze the impact of e-leadership on teachers’ attitudes through technological self-efficacy. However, it has the same limitations as the previous research. Therefore, the findings of our study can fill an important knowledge gap.

According to Contreras et al. (2020), leaders are obliged to adapt to the new conditions proposed by virtual work environments, through additional skills. Under this perspective, beyond having charisma, ethical leaders influence others through example and are clear transmitters of emotional skills that are essential to face disruptive and volatile scenarios. Their resilient and natural character makes them credible and horizontal persons seeking legitimacy through actions far from self-interest (Javed et al., 2018). Undoubtedly, ethical leadership seeks a vertical and transversal distribution of responsibilities, with the help of joint reflections and with a constant transfer of authority (Duthely, 2017). In fact, the ethical leader communicates clear ideals that prevent opportunistic behavior in remote work environments. Thus, moral principles are basic to address problem resolution from an ethical perspective (Elkington, 2020).

Additionally, telework raises moral concerns such as information excesses that tend to overlap with family and professional life (Kanwal & Isha, 2022). An ethical leader can solve these by preventing imbalances that harm employees (Darics, 2020). This management style, by nature, combines several beneficial traits. These include, for example, benevolence, integrity, joint decisions, and fair treatment, and it is an essential element to address the transition from an on-site environment to an online one (Figueiredo et al., 2022; Segbenya & Okorley, 2022). Simultaneously, the concern of ethical supervisors for their followers keeps a common psychological connection that prevents any hint of psychological isolation related to telework (Afshar Jahanshahi et al., 2022; Saha et al., 2020). Ethical leaders guide employees, and this constant support has a positive impact on their conviction to successfully achieve a determined objective (Ren & Chadee, 2017). It is probable that sustained feedback and precision in the information provided are two essential characteristics of e-leadership (Toleikienė et al., 2020). Therefore, ethical leaders become a guarantee for followers to express their concerns and jointly seek honest solutions in a trust framework.

The transfer of valuable resources between leaders and followers limits emotional concerns from possible threats, which ultimately increases self-efficacy (Ashfaq et al., 2021). Finally, ethical leadership is characterized by decentralized and democratic management, which results in effective administration (Latta & Clottey, 2020). In fact, technology transforms it into e-leadership that encourages performance by minimizing the digital distance through open communication, idea exchanges and trust development (Contreras et al., 2020; Lee, 2009). Consequently, the following hypothesis is proposed:

**H2.**  *Ethical leadership is positively related to followers’ self-efficacy in virtual work environments.*

### 2.3. Ethical Leadership, Egoistic Ethical Climate, and Self-Efficacy

Some ethical climates, such as benevolent or principled, have been widely analyzed. In contrast, a climate based on selfish attitudes, such as self-interest or organizational interest, requires more research. A selfish or instrumental criterion is based on the moral philosophy of egoism, which implies that the dominant consideration in the ethical reasoning process will be what is most beneficial for the individual. In this regard, Murphy and Free (2016) establish a relationship between instrumental climate and a malicious work environment linked to fraud. Similarly, De Hoogh et al. (2021) suggest that an instrumental climate increases the tendency for certain leadership styles to exhibit negative behaviors, such as abusive supervision. Finally, Tziner et al. (2015) find that a person-oriented egoistic ethical climate is negatively related to the perception of organizational justice, as it hinders personal goals. Therefore, most previous research highlights the negative impact that an egoistic ethical climate has on organizational attitudes. However, as far as we know, no previous article has analyzed the role of an egoistic ethical climate in a virtual work environment, which could represent a significant advancement in the study of some ethical climates that seem to be detrimental, at least from a common interest-oriented perspective.

A systematic breach of the norms that regulate an organization is considered, in general terms, deviant and unethical conduct that relates to self-interest (Renn et al., 2005). However, breaking the rules can also be linked to the will to support the organization so that it fulfills its purposes (Morrison, 2006). In this direction, a high perception of self-efficacy can maximize individual confidence to successfully execute a given task. Therefore, individuals may focus their attention on the potential benefit of their behavior and may not try to understand the ethical risk associated with it (Vardaman et al., 2014). In fact, egoistic climates encourage decision-making based on self-interest and organizational interest. This tends to design work environments where rivalry fosters many initiatives that might go against prosocial norms (Swanepoel et al., 2015).

On the other hand, ethical climates significantly affect individuals’ identification with the organization. Hence, ethical leadership seeks to build an environment that directs its efforts toward a common interest and concern for others—in other words, towards benevolent environments with specific regulations (Gumusluoglu et al., 2020). Under this light, an egoistic climate tends to weaken emotional ties and identification among organization members (Cheng & Wang, 2015). Measures promoted by ethical leaders have the goal of fostering mechanisms that improve moral behavior, and go beyond personal interests, as they prioritize the needs raised by followers (Ozavize Ayodele et al., 2019). Thus, an organizational climate justified by self-interest can continuously hinder and deteriorate ethical prerogatives until these lose their scope (Morris, 2016).

The social cognitive theory is a useful tool to clarify this situation. Self-efficacy is sustained, and increases based on four main aspects: achievement experience, indirect learning, positive feedback and stable emotions (Bandura, 1986; Bandura et al., 2001). In practice, self-efficacy is conditioned by the control of individuals over the performance of a behavior, and it is stable when this self-regulation is independent of any external factor (Lumpkin & Achen, 2018). However, self-efficacy is categorically affected when external forces limit the behavioral domain (Brändle et al., 2018). Consequently, when an ethical leadership style has to endure an egoistic climate to influence followers’ self-efficacy, its characteristics decline until they are invalid and, on the other hand, self-interest prevails. This means individual competences and interests, organizational success, and emotional self-sufficiency (Gruber & MacMillan, 2017).

When job alternatives completely ignore collective needs and focus on maximizing a line of behavioral reasoning that prioritizes personal gain and selfishness, a fracture results, leading to moral disconnection (Fida et al., 2018). This enables deactivating any acceptable ethical principle. In other words, there is a superior morality supporting what is useful to oneself above any reasonable criteria related to what is just and upright. Ethical leadership and job self-efficacy are gradually dissociated under this scenario until they are completely separated. Therefore, the following hypotheses are proposed:

**H3.**  *An egoistic ethical climate positively relates to individual self-efficacy in virtual work environments.*

**H4.**  *An egoistic climate inversely moderates the positive relationship between ethical leadership and self-efficacy, in virtual work environments. The greater the perception of the egoistic ethical climate, the less positive the influence of ethical leadership on individual self-efficacy.*

### 2.4. Research Model

**Figure 1:**
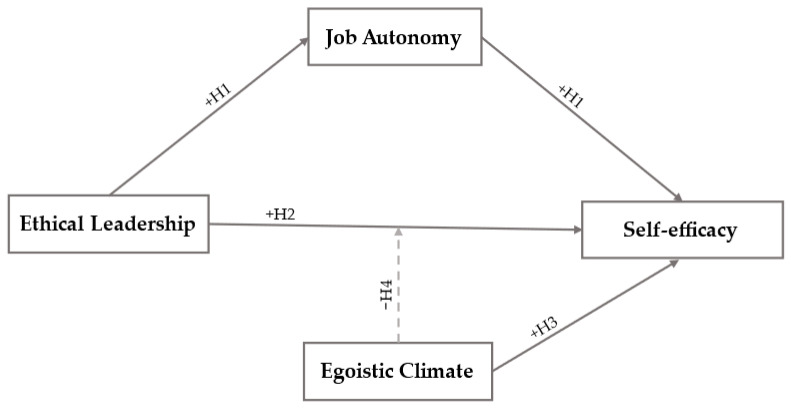
Research model.

The positive impact of ethical leadership and an egoistic ethical climate on self-efficacy represents a significant advancement in the existing knowledge on leadership and ethical climates. Additionally, the inverse moderating effect of an egoistic ethical climate indicates that the values the ethical leader tries to convey are incompatible with a climate whose main moral reasoning is individual over collective. Finally, job autonomy is a mechanism capable of explaining how or why a relationship between ethical leadership and self-efficacy is established. Figure 1 shows the research model and the positive or negative direction of the four proposed hypotheses.

## 3. Methods

### 3.1. Participants

Our sample was made up of 448 telecommuters employed in the Colombian electricity sector. Specifically, they worked for six companies with offices in Bogotá, Cali, Medellín, Manizales and Pereira. Sampling was performed probabilistically, by conglomerates considering the main cities of the country. The response rate was 100%. Regarding gender, 175 (39%) of the participants were women, and 273 (61%) were men. The average age was 37.18 years (SD = 10.059; range: 20–69). A total of 364 employees had permanent contracts (81.25%), and 84 had temporary work contracts (18.75%). The mean seniority was 13.06 years (SD = 8.82; range: 1–38 years). Regarding occupation, 86.6% (308) were professionals, 8.9% (40) held intermediate jobs, and finally, 4.5% (20) were managers. All of those surveyed had a university-level education, while 57.4% (257) completed graduate studies. A total of 42% (188) did not have children.

### 3.2. Instruments

Control Variables: Educational level, organizational position, seniority and gender were used as control variables. It is possible that employees with high adaptation to organizational idiosyncrasies are more autonomous and self-effective. Additionally, educational level is likely to be positively related to job autonomy and self-efficacy. In fact, job autonomy tends to increase as the level of formal or informal education increases. Similarly, it seems logical to think that a person with a greater number of educational resources will have more skills to solve their daily tasks more effectively. Finally, we expected that the organizational position will be another factor actively related to job autonomy and self-efficacy. To measure permanence, survey participants were asked to indicate how long they had been working using a minimum scale of 0 to 1 year. Gender was coded as 0 for men and 1 for women. Educational level was measured by considering whether the respondent had a bachelor’s or engineering degree, specialization, master’s degree, or doctorate. The organizational position was divided into four categories: professional, specialist, coordinator, and area director.

Ethical Leadership: A one-dimensional scale proposed by Brown et al. (2005) was used, composed of 10 reagents and with a Cronbach’s Alpha of 0.94. It was initially used with a scale of 7 options. This construct was used by Feng et al. (2018) with a 7-level Likert scale and a Cronbach’s Alpha of 0.91. The perception of organizational leadership was measured through actions, interpersonal relationships, and communication, among other characteristics, to determine if they transmitted trust and aligned with ethical behavior.

Job Autonomy: The one-dimensional scale designed and proposed by Spreitzer (1995), with three reagents and a Cronbach’s Alpha of 0.72, was used; this was used by Santiago-Torner (2023e), with a Cronbach’s Alpha of 0.89. It values if an employee has enough freedom to be able to decide in their job and exercise some control over it.

Self-efficacy: The 6-item unidimensional scale suggested by Schaufeli et al. (1996) was used, assessed through a 4-point Likert scale and with reliability between 0.76 and 0.90. This was used by Salanova and Schaufeli (2000) with an α of 0.80. It evaluates skill and capacity to successfully achieve an objective.

Egoistic Ethical Climate: Part of the multidimensional scale proposed by Victor and Cullen (1988) was used. Provided is dimension number 1, which is the center of analysis, where the individual, the local and the cosmopolitan coexist together, with the egoistic criterion belonging to dimension number 2. This is composed of 14 items in three subscales: self-interest (7 questions), business benefit (3 questions) and efficiency (4 questions). The way to perfect individual interest is evaluated above all other considerations. This scale was used by Santiago-Torner (2023b, 2023e) with a 6-point Likert scale and an internal consistency of 0.77. The initial scale uses 5 Likert points and shows a Cronbach’s alpha (α) between 0.69 and 0.85.

To avoid a possible conceptual and methodological misalignment in the measurement of egoistic climate, we followed the proposal of the original authors Victor and Cullen (1988). Conceptually, ethical climate is a collective construct based on the perceptions of the members of an organization. However, Cullen et al. (2003) and Victor and Cullen (1988) measure egoistic ethical climate through individual perceptions. For example, Cullen et al. (2003) use two different groups. The first includes respondents from seven departments of a telephone company, and the second includes respondents from four accounting organizations. Similarly, Victor and Cullen (1988), when they first analyze egoistic ethical climate, do so through the individual perception of 33 employees of a printing company, 450 employees of a savings bank, 500 employees of a telephone company, and 200 managers of a manufacturing plant. Considering the response rate of the different surveys, in the end, only the individual perceptions of 872 employees were taken into account.

Following this same methodological line, we reviewed other more recent articles. For example, Saleh et al. (2022) measured ethical climate through 260 individual perceptions from workers in nine industrial manufacturing SMEs in Selangor (Malaysia). On the other hand, in the research by Hamoudah et al. (2021), they measured ethical climate through the individual perception of 419 civil servants from Malaysia and the Kingdom of Saudi Arabia. Finally, Mazharul Islam and Alharthi (2020) used the Victor and Cullen (1988) scale to measure egoistic ethical climate. The ethical climate questionnaire was answered by 250 senior executives and general managers of SMEs located in Saudi Arabia. That is, their results were based on individual responses that, in turn, shaped the respondents’ perception of the ethical climate surrounding them.

### 3.3. Procedure

All the proposed research passed the Ethics Committee of the University of Vic—Central University of Catalonia in July 2021 with codes 2021 and 170. The information was collected during the last four months of 2021. Different privacy agreements were determined in an initial phase and all the study materials were sent to the organizations: objectives, data protection security, participation description, and voluntary withdrawal option with the corresponding document to complete, among others. The project began in April 2021, when it was presented to close to 40 companies in the sector at an annual event aimed at promoting leadership and ethical climates as buffers against possible irregularities. The Colombian electricity sector is characterized by its constant desire to generate transparency in results, agreements, associations, etc. The six companies involved in the study represent the sector, as they are subsidiaries of large multinational companies that make up the Colombian electricity community.

## 4. Data Analysis

Revised in the first phase using the Hotelling test (T2), in the variables observed, are univariate and multivariate outliers, and no outliers were found. Regarding the normality of the variables, asymmetry, and kurtosis values below 2 and close to 0 are sought, which denotes normality according to Kline (1998). Complementarily, a test of homogeneity of variances is conducted through Levene’s statistic and from the results, *p* > 0.05, homoscedasticity is determined. The SPSS v.25 statistical program is used.

**Figure 2:**
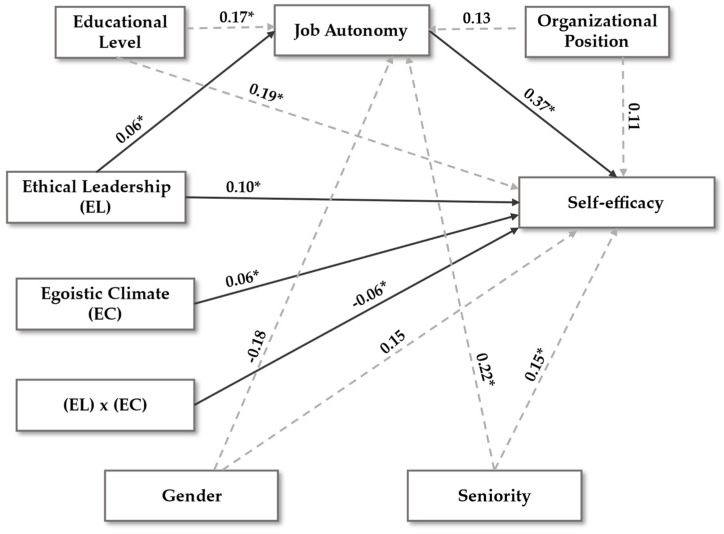
Includes the value of the regression coefficients calculated for each of the variables studied. Regression analysis. Non-standardized coefficients. * *p* < 0.05.

**Figure 3:**
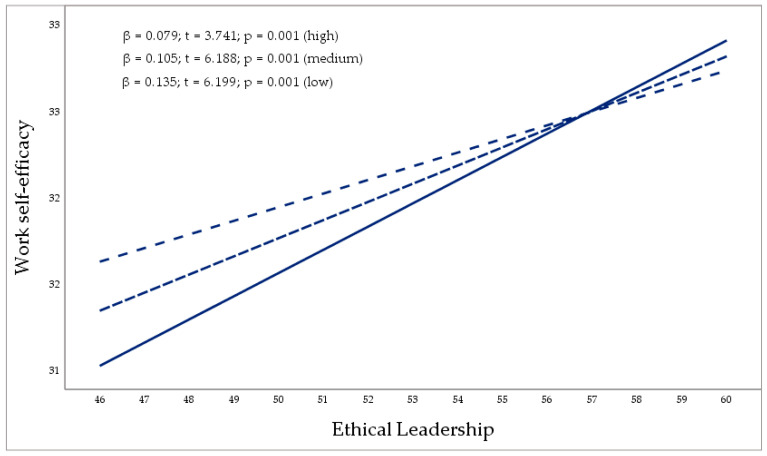
Moderation of the egoistic ethical climate (low, medium, and high perception) on the relationship between ethical leadership and self-efficacy. The greater the perception of an egoistic ethical climate, the lesser the influence of ethical leadership on follower self-efficacy.

Descriptive statistics and correlations between study variables were calculated in the second phase (Table 1). Likewise, model relevance was evaluated through convergent and discriminant validity (Table 2). Subsequently, multiple regression analyzes were conducted with PROCESS v.3.5 macro (Hayes, 2018) to study the moderating function of the egoistic ethical climate variable (W), along with the mediation of job autonomy (Mi), regarding the relationship between ethical leadership (X) and job self-efficacy (Y) (Table 3). Model 5 (mediation and moderation) is used for this complex function with a confidence interval of 95% and a total of 10,000 bootstrapping samples. The collinearity problem is avoided by determining the Variance Inflation Indices (VIF) that are below 5 (Hu & Bentler, 1999). The model required for this analysis is built alongside with the AMOS v.26 macro (Hayes, 2018) (Figure 1). Finally, the Johnson–Neyman technique is used to specify the areas of statistical significance, which enables seeing the conditional effects of an egoistic ethical climate (W) regarding ethical leadership (X) − job self-efficacy (Y) (Figure 2 and Figure 3).

To review the possible differences between the samples, the effect size was evaluated. In this regard, the parametric statistical test Student’s *t*-test is used, and the effect size is evaluated using Cohen’s delta test. A value close to 0.20 indicates that the statistically significant differences are small. A value close to 0.50 indicates that the statistically significant differences are medium. Finally, a value close to 0.80 indicates that the statistically significant differences are large. Firstly, when analyzing the different independent samples, the results indicate that men and women do not present statistically significant differences regarding the perception of job autonomy or self-efficacy. Secondly, we analyzed job tenure through the following comparisons: (0–1, 1–3); (4–6, 7–9) and (10–13, +13), concluding that the years worked in the organization do not present statistically significant differences regarding the perception of job autonomy and self-efficacy. Finally, educational level and organizational position also do not present statistically significant differences regarding the perception of job autonomy and self-efficacy.

## 5. Results

Table 1 presents the number of items per scale, means, standard deviations, and bivariate correlations. The significant association between job position and job autonomy stands out in this table. In fact, ethical leadership plays an active role in incorporating autonomy as a basic aspect of work within the organizational context, and it is logical that a higher influence status leads to a wider range of autonomy.

The proposed model was verified using the process suggested by Chin (1998). The following analyses were performed to confirm the solidness of all variables: composite reliability (CFC), average variance extracted (AVE), and discriminant validity (DV). Similarly, the critical coefficients (CR) fit the recommendations of Hair et al. (2011) (>1.96; *p*-value less than 0.05). CFC and Cronbach’s Alpha values are above 0.70, which ensures the reliability of the constructs used. The AVE factors are between 35 and 79%, which is significant (Bagozzi et al., 1998). The square root of AVE must be greater than the Pearson correlations between variables to have discriminant validity, which clearly occurs (Fornell & Larcker, 1981). In fact, the smallest square root is 0.59, and the largest correlation is 0.37 (see Table 2).

Table 3 specifies the mediation and moderation analyses with non-standardized regression coefficients. The coefficient of determination (R^2^) explains 32.8% of the variance of the self-efficacy-dependent variable. Four control variables, gender, seniority, educational level and organizational position, are used to give solidness to the model and to the results obtained.

The analyses were performed considering higher and lower values (LLCI and ULCI) as dimensions. Zero (0), when present within these ranges, defines the invalidity of a regression analysis.

Ethical leadership (X independent variable) is related to job autonomy (mediating variable) through the route ai (β = 0.059; *p* < 0.05; [0.023, 0.078]). Job autonomy relates to self-efficacy (Y dependent variable) through the bi route (β = 0.369; *p* < 0.05; [0.228, 0.456]). Furthermore, the indirect effect of job autonomy on the relation between ethical leadership and self-efficacy is positive (β = 0.045; *p* < 0.05; [0.017, 0.080]). Concluded from this first part of the analysis is that job autonomy fulfils its mediating function; therefore, H1 is supported.

The second part of the analysis corresponds to the moderation process, when an egoistic ethical climate (moderating variable) influences the relation between ethical leadership and self-efficacy. The three direct conditional effects indicate that ethical leadership gradually loses its positive influence on self-efficacy as the perception of an egotistical climate increases: low effect (47)—(β = 0.135; *p* = 0.001; [0.092, 0.177]); medium effect (56)—(β = 0.105; *p* = 0.001 [0.072, 0.139]); high effect (64)—(β = 0.079; *p* = 0.001; [0.038, 0.121]). Simultaneously, route c3’ (β = −0.061; *p* = 0.034; [−0.013, −0.001]) confirms H4. Therefore, egoistic climates inversely moderate the positive relationship between ethical leadership and self-efficacy in virtual work environments. The greater the perception of an egotistical ethical climate, the less positive the influence of ethical leadership on individual self-efficacy.

Finally, the third part of the analysis corresponds to the direct effect of ethical leadership on self-efficacy. Therefore, route c1’ (β = 0.104; *p* < 0.05; [0.117, 0.459]) verifies H2. Furthermore, the analysis of the c2’ routes (β = 0.056; *p* < 0.05; [0.025, 0.346]) confirms H3. In other words, egoistic climates relate to self-efficacy.

Figure 3 graphically reproduces the moderation process of the egoistic climate variable (W) considering the relation between ethical leadership (X) and job self-efficacy (Y), respectively. PROCESS provides three scores for the variable (W) considering the mean score (±1) of its standard deviation. The values provided are low, medium, and high, and, respectively, coincide with the following scores: 47, 56 and 64. Effects 1, 2 and 3 specify that the greater the perception of W, the lesser the effect of X on Y.

**Figure 4:**
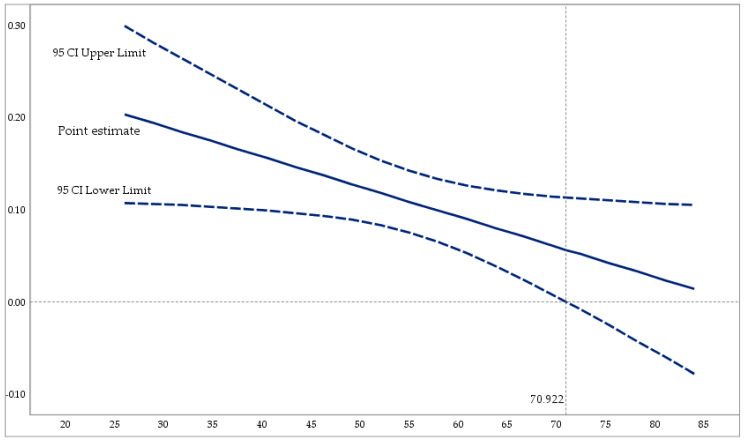
Conditional effect of ethical leadership on job self-efficacy, depending on the different values of the moderating variable (egoistic ethical climate).

Figure 4 represents the conditional influence of ethical leadership (variable X) on job self-efficacy (Y) with the three values of the moderating variable ethical climate (W). The Johnson–Neyman technique is used to define the zone of relevance of the conditional effect. Figure 3 highlights its importance in the upper left quadrant. Thus, W is important up to 70.922. A total of 69.3% of the sample is in this segment.

## 6. Discussion

The first hypothesis tested in this study is the useful mediation of job autonomy, regarding the relationship between ethical leadership and individual self-efficacy. This intermediation effect follows two theoretical assumptions. The first verifies the affinity between ethical leadership and job autonomy, which coincides with Frazier and Jacezko (2021), Liu et al. (2020) and Santiago-Torner (2023a), among others. Ethical leaders have a clear inclination to build work contexts where followers can freely choose a self-management model, through alternative and independent forms of behavior. In fact, ethical leaders are shifting away from the conventional scheme where work environments depend on a constant behavioral orientation (Kalshoven et al., 2013).

In fact, ethical leaders propose a continuous exchange with followers to establish shared responsibility guidelines in the achievement of objectives. Therefore, job autonomy, far from becoming a mechanism that interrupts contact, becomes a source of mutual guidance that stimulates performance and self-efficacy (Liu et al., 2020). Additionally, the option of sharing workloads indicates maturity in the relationship between leaders and followers and promotes stable support and development of two-way initiatives. Ethical leadership naturally models an organic environment where interpersonal relationships are part of the values transmitted. Autonomy, in this context, becomes a priority tool contributing to organizational efficacy through proactive and persistent approaches (Santiago-Torner, 2023f). When ethical leaders assume the role of e-leaders, discipline and autonomy are fostered as essential components that simultaneously improve followers’ well-being and results (Contreras et al., 2020).

The confirmation of the first hypothesis significantly contributes to the existing literature, as it not only fills an important knowledge gap but also adds to the set of positive results that various authors have attributed to ethical leadership (Islam et al., 2024; Tetteh et al., 2024). Previous studies have shown that ethical leaders give meaning to work tasks through normative standards that consider the employee’s role and the impact of work on their lives, both within and outside the organization. In fact, these studies have demonstrated that ethical leaders improve the nature of work and the perception of autonomy (Liu et al., 2020). However, attention had not been paid to the role of the ethical leader in a virtual work environment and, more specifically, to its possible impact on job autonomy and self-efficacy.

The second theoretical assumption that confirms the mediation process is that job autonomy and self-efficacy are significantly related. This is particularly consistent with Wattoo et al. (2020) and Dedahanov et al. (2019). Bakker et al. (2011) state that job autonomy and self-efficacy are part of the work, and personal resources employees have to face the difficulties and obstacles of work itself. In fact, job autonomy gives followers the possibility of intentionally redistributing tasks, which increases their individual capacity to set goals and overcome work inconveniences more easily; that is, it increases their level of self-efficacy (Wattoo et al., 2020). Thus, and according to an updated approach of the social learning theory (Rumjaun & Narod, 2020), acquiring a skill requires certain transitions in external behavior. These are only perfected using observation, retention, and repetition, along with progress in cognitive processes. Therefore, employees with increasing autonomy have more options to notice the effects and advances of their own actions, even when irregular, Dedahanov et al. (2019) compared to employees with less work flexibility. Finally, in virtual work environments, autonomy and self-efficacy act as potential mitigators of the stressful effects of work overloads on followers’ emotional health. In other words, they assume the role of a work resource (Mihalca et al., 2021).

Another argument verified by this article is that ethical leadership opportunely manages an environment where job self-efficacy tends to grow. Therefore, the greater the perception of this management style, the better the performance of followers, in agreement with Ren and Chadee (2017) and Walumbwa et al. (2011). Bandura et al. (2001) specify four methods to optimize self-efficacy, specifically: vicarious or modeled experience, verbal persuasion, affective activation, and personal achievements. Considering this, ethical leadership affects these four points through the theory of social learning (Walumbwa et al., 2011). The leader, as a moral person, acts as an ethical model. This means that leaders have certain desirable aptitudes that followers want to reproduce and incorporate into their own life. Likewise, ethical leaders insist on the importance of making decisions through moral convictions (Chikeleze & Baehrend, 2017) and emphasize the critical role of followers to achieve important goals (Bai et al., 2019). From this angle, employees acquire the ability to strategically analyze, and this complex process enhances their self-efficacy. Therefore, followers advance through observation and imitation of the leader and also establish a cause-effect relationship that directs their behavior based on the credibility of the model to imitate (Ren & Chadee, 2017; Santiago-Torner, 2023d).

In fact, the benevolent stance of ethical leaders places followers in an ideal position where they can progress and correct their perception of self-efficacy, within a context clearly marked by relationships of trust. Consequently, employees lean toward a convincing moral prototype (Su et al., 2021). Ethical leaders, on the other side, as safe sources of feedback, promote reactions that extend the signals of self-efficacy in employees beyond results (Ashfaq et al., 2021). They focus on how to proceed to reduce tension and intensify reflection and self-efficacy, which awakens feelings of affection in employees. Finally, ethical leaders show sincere interest in their followers. Therefore, ethical leadership builds a work environment in which followers feel emotionally safe. This climate of trust inspires, among other things, a perception of achieving personal goals through greater self-efficacy (Bai et al., 2019). The relationship between leadership and performance within a virtual work environment is, of course, conditioned by identification between leader and follower (Contreras et al., 2020). Consequently, ethical leaders, when having the main aspects needed by e-leaders—specifically trust, continuous communication, and cooperation (Elyousfi et al., 2021)—arrive at a virtual management style that goes beyond basic skills and can transform the result of followers’ work through a new concept of technological self-efficacy (Weerawardane & Jayawardana, 2022).

The verification that job autonomy is related to self-efficacy is an especially important finding. A remote work environment that proposes positive strategies to empower employees becomes a resource that, in addition to improving job autonomy, enhances the employee’s sense of control and self-assessment to be more effective. In fact, self-efficacy is considered an important personal resource that improves overall stress resistance, is associated with higher levels of resilience, and promotes positive coping strategies in response to environmental demands (Mihalca et al., 2021).

The third hypothesis corroborated by this article is that an egoistic ethical climate can positively influence self-efficacy. This is consistent with the results obtained by Swanepoel et al. (2015) and Tanner et al. (2015). People in egoistic ethical climates have productivity and organizational benefit as their main interest, and, therefore, it is likely that breaking formal rules is not a concern (Vardaman et al., 2014). Actually, an instrumental climate builds a competitive work environment where individual determination to achieve goals depends on an uninterrupted effort able to handle difficult circumstances, with a strong sense of self-efficacy. In fact, Brändle et al. (2018) identify that people who are particularly driven by self-interest have highly competitive capacity and are more self-efficient as their vision of a successful professional converges with a high perception of competence. Therefore, self-efficacy is related to personal beliefs and to a reflection of the confidence individuals have to be able to coordinate their abilities and skills to achieve goals with high performance (Santiago-Torner, 2023c; Swanepoel et al., 2015).

The main finding in this research is that an egoistic ethical climate, as a moderating variable, systematically obstructs and attenuates the influence of ethical leadership on job self-efficacy, until this relationship is deactivated. This result fills an important knowledge gap, as it explains the circumstances under which ethical leadership loses its usefulness. This can lead to valuable practical implications. Similarly, no contrasting studies with a similar model have been found, which consolidates the importance of this analysis. Ethical leaders are essential pieces in the development of an ethical climate (Mayer et al., 2010). Thus, their two moral facets—person and manager—seek the common benefit through a shared perception of integrity, justice and ethical standards (Al Halbusi et al., 2021).

Specifically, the character of ethical leaders is sustained by virtue of basic principles such as: responsibility, two-way communication, common feelings of trust, and clear moral guidelines regarding what is perceived as right or wrong. Consequently, ethical leaders transcend self-interest to focus on what is organically beneficial (Ozavize Ayodele et al., 2019; Özden et al., 2019). Hence, their transactional efforts clash with egoistical climates. Moral arguments seeking balance solely within oneself are likely to hinder ethical procedures through egoistic behavior. Certainly, an organizational climate cannot be extremely ambivalent. In other words, it cannot predominantly maximize its own interest while simultaneously promoting collective benefits. Therefore, an egoistic climate can distort the norms related to civic management and induce unethical behavior. In fact, Gorsira et al. (2018b) conclude that an egoistic climate, when having certain organizational hegemony, inclines ethical decisions towards self-interest as the main consideration. This is the reason why employees perceive that behaviors with an individual emphasis are accepted as correct and do not avoid what is amoral. In other words, personal arguments, regardless of their nature, become factors that negatively condition all corporate conduct.

In this context, individual and organizational egoism point to an institutional tension leading to work dissatisfaction, frustration, and low loyalty (Atshan et al., 2022). Therefore, an organizational climate based on self-interest can decrease the attraction of employees, with a high sense of self-efficacy, towards critical challenges and complex tasks (Asif et al., 2022). This in turn disables the role of ethical leadership. Consequently, egoistic principles are occasionally perceived as destructive because decisions can imply potential harm to others, and they also question or deteriorate existing standards and rules (Manning, 2020). In conclusion, injustice and dehumanization are predominant feelings that represent a setback in job self-efficacy, as a useful response to adverse situations. This scenario obscures the role of ethical leadership, as its essence tries to improve individual efficacy through constant feedback on performance and a clear perception of fairness (Bashir & Hassan, 2020), which egoistic ethical climates halt and even reverse.

## 7. Theoretical Implications

Ethical leadership is an essential element for the functioning of an organization, as it has a direct impact on people’s actions and behaviors. In fact, ethical leadership can become a valuable resource that enhances individual autonomy and self-efficacy. However, to our knowledge, there is no research establishing a relationship between ethical leadership and self-efficacy taking into account a mediating process (job autonomy) and a moderating process (egoistic climate). Therefore, our findings are particularly important and contribute significantly to the existing knowledge on the effect of ethical leadership. In addition, our model of mediation and moderation has another differential aspect. Specifically, it is controlled by four variables, which strengthens the results obtained and their reliability. Secondly, this study integrates ethical leadership and virtual work environments responding to previous calls in the literature about exploring the influence of specific leadership styles through information technologies.

## 8. Practical Implications

Individual preferences for cooperation appear to act as dynamic factors that eradicate ethical deviations in the workplace (Pletzer et al., 2018).

In this sense, organizations can prevent the presence of unethical behavior through a selection process that prioritizes prosocial values such as equality or equity. In fact, Curran et al. (2019) suggest critical incident interviews as an effective tool to identify and measure these key competences, as they enable extracting delimited knowledge and attitudes, and eventually find out where interviewees specifically direct stimuli and their extent of alignment with a particular ethical climate.

Prosocial motivation refers to the will to guarantee and increase the well-being of other people, which improves employee commitment, persistence in achieving objectives and consequently boosts individual self-efficacy (Li, 2019). Therefore, employees with socially focused principles act as moral agents who stand in solidarity with ethical leaders and establish shared synergies with them to achieve challenging tasks (Arshad et al., 2021). Generally, social actions are not born voluntarily and depend on the influence of an ethical leader, through trustworthy attitudes and behavior (Charoensap et al., 2019).

Thus, promoting an ethical management style that encourages high-quality interpersonal relationships with followers is essential to guarantee a climate aimed at group learning. Frequently, service-oriented activities strengthen organizational citizenship behavior (OCB) through the bond of mutual attention established between leaders and followers (Babič et al., 2019).

In Colombia, specifically in its electricity sector, an ethical approach to leadership is key for different reasons. This industry segment is usually public. Therefore, it is dedicated to serving the neediest social spheres (Santiago-Torner & Rojas-Espinosa, 2021). Additionally, ethical leadership affects the behavior of followers who move and impact outside organizational limits. Therefore, individuals with high OCB and prosocial attitudes will help reconfigure a country where 40% of the population lives on the verge of extreme poverty.

At the same time, Colombia is a country with high levels of corruption and with one of the greatest social inequalities in the world (Páez & Salgado, 2016). Consequently, it is necessary for the Colombian industrial fabric to look at ethical climates such as the benevolent or normative ones, as the egoistic ethical climate is clearly aligned with behaviors that prevent what is moral, or where ethical conceptions acquire a secondary role (Gorsira et al., 2018b). Additionally, this research has shown that a climate that leads to self-interest or organizational interest disables the role of the ethical leader. Considering that employees are the critical aspect to consider when promoting ethical behavior, inside and outside the workplace, it is not possible to do so without this management style (Santiago-Torner et al., 2024a, 2024b). Hence, Colombian organizations, beyond good will, need to grow ethically through two different paths: hiring new ethical leaders and training existing ones. These represent a challenge, as 74% of the population surveyed has extensive seniority and is likely anchored in customs that tend to covertly regulate their day-to-day life. Therefore, change requires the implementation of a system that rewards the ethical and severely disciplines the amoral.

From a perspective of promoting well-being at work, autonomy cannot have the opposite effect of what was initially intended. In other words, organizations without a defined collective structure tend to distort the positive effect of autonomy through work rhythms that extend the work shift, instead of restricting it. Therefore, it is a priority to consider the multidimensional nature of tasks and the complexity of timelines that establish limits and collective temporary structures. In addition, self-efficacy also depends on including truly applicable shared concentration spaces and active breaks (Väänänen et al., 2020).

Our results suggest specific ways to counteract the harmful effects associated with a self-interest climate. For example, organizations can establish clearly defined policies in their selection processes, incorporating employees who are not only self-efficacious but also naturally resistant to the influence of personal incentives on their ethical thinking. In other words, self-interest should not be a key characteristic of their personality. In fact, there are various psychometric tests that assess emotional intelligence and service orientation. Additionally, organizations can implement mechanisms to identify employees with high self-interest, such as personality tests or assessments of egocentric attitudes. These employees can receive special guidance from their leaders to develop their ability to help others. Altruistic behaviors minimize the potential harm of an egoistic ethical climate.

Self-interest that leads to an egoistic climate is a source of motivation that explains much of personal success. However, when there is no control over self-interest, personal desires are likely to cross certain ethical boundaries. Therefore, an egoistic ethical climate is not necessarily associated with negative consequences and can have different impacts on a person’s confidence in their own abilities to achieve a goal (Wu et al., 2021). In fact, the influence of the ethical leader can become a turning point that balances the opportunities for selfish gains and the preservation of certain ethical standards that prevent moral disengagement.

Unethical behavior results from the failed activation of self-regulation processes. People internalize standards of conduct, such as social and organizational values, by imitating inspiring behaviors. The theory holds that individuals, when faced with unethical behavior, activate moral standards and self-regulation mechanisms to self-censor their actions (Khozin et al., 2024). In this sense, the ethical leader, through words and actions, transmits constant moral signals that guide the employee to establish boundaries between right and wrong (Hosseini & Ferreira, 2023). Therefore, a first implication to reverse the negative moderating effect of an egoistic ethical climate is to avoid reward systems centered on personal gain. Incentivizing high performance and self-efficacy can trigger morally disengaged reasoning. From this perspective, a potentially powerful force that counteracts the effects of personal gain on moral disengagement is being aware of the situational harm an action can cause to others (Kish-Gephart et al., 2014). The ethical leader seeks to build trust-based relationships with the follower. Trust is a fundamental element that conveys responsibility and influence (Islam et al., 2024). Therefore, a meaningful relationship between leader and follower can lead to empathetic responses that reconcile self-interest and self-efficacy through a strong social and ethical commitment. In fact, principles of loyalty are values that define the quality of a relationship and the way to support a person or organization when faced with difficulties or incompatibilities in a moral dilemma (Agu et al., 2024).

Secondly, conscientiousness is a personality trait that can become a compensatory force at the individual level to avoid the moderating effect of an egoistic ethical climate. Conscientiousness refers to the degree to which individuals are reliable, hardworking, and organized. In addition to its strong relationship with self-efficacy, conscientiousness is also believed to have a moral component (Sadat Mousavi & Ebrahimi, 2024). Conscientious individuals experience a high degree of moral obligation, value truth and honesty. They also maintain a high respect for duties and responsibilities, are self-disciplined, and have a strong sense of self-control. Taken together, these characteristics suggest that when a conscientious individual faces a great opportunity for personal gain, they will have the tools to avoid deactivating their moral standards; that is, they will be able to balance individual interest with potential ethical repercussions.

Different studies have associated ethical leadership and conscientiousness, such as Babalola et al. (2019), Rice et al. (2020), and Saleh et al. (2022). Ethical leadership provides the foundation for employees to perform their tasks effectively and for that effectiveness to meet their psychological needs. Additionally, the influence of the ethical leader facilitates the follower’s communication of any errors or confusing situations they encounter (Pakizekho & Barkhordari-Sharifabad, 2022). Therefore, when the leader focuses on promoting not only the organization’s norms and values but also certain personal characteristics, such as conscientiousness, they establish effective links for the employee to assume greater responsibility for their duties and objectives from an ethical perspective, which can prevent self-interest from prevailing and distorting its impact.

## 9. Limitations and Future Research

This study is not exempt from limitations. First, it is based on a self-observation survey; therefore, this can translate into social desirability bias (Donaldson & Grant-Vallone, 2002). Likewise, the principal researcher was present in all the surveys and, when communicating the research and its objectives, expressed the importance of responding rigorously, as the validity of the results lies on that. Additionally, a second limitation, cross-sectional studies cannot accurately determine a temporal sequence between variables as the assessment is synchronous. Finally, the possibility of a longitudinal study increases, as it can more clearly corroborate the causality of the results.

Regarding future research, few authors have analyzed the relationship between the different ethical climates and job self-efficacy (Swanepoel et al., 2015), and none of these authors, at least within the bibliographical review in this article, use its multiple dimensions with a moderating character to understand, more broadly, under what circumstances ethical leadership activates or neutralizes followers’ self-efficacy (Santiago-Torner, 2024; Santiago-Torner et al., 2024c). Therefore, it is possible to reproduce the model used in this research in other ethical climates, such as benevolent or normative, which can initially boost the effects of ethical management, as they are more similar in their characteristics. Additionally, remote work has been a contextual aspect on this occasion, and it is a priority to specifically know how ethical leadership styles relate to telework and its characteristics, as only Lee (2009) establishes some kind of relation.

On the other hand, the cross-sectional study design may not fully explain the causal relationship between the study variables; however, rather than devaluing the current results, it is suggested that in the future we follow up by repeating the study or using a longitudinal study design to test the results of our causal relationship. Finally, this research focused on the Colombian electricity sector; therefore, future research may address other energy sources such as mining or hydrocarbons.

## 10. Conclusions

Ethical leaders promote a collaborative management style. They empower followers by facilitating the control of their own work. Hence, a stable perception of support transmits enough confidence and autonomy for employees to persist and intensify efforts, which improves their sense of self-efficacy and allows them to challenge the limits of any activity, executing it successfully. Actually, ethical leaders and followers develop each other through constant reflections along with shared adjustment and observation mechanisms that, in addition to improving efficiency, create a climate that processes moods adequately (Chughtai, 2015).

Ethical leadership drives greater individual self-efficacy from several perspectives. Exchanging valuable resources probably increases trust between leaders and followers, as both perceive that their paths coincide in an honest process (Malik et al., 2023). Undoubtedly, both establish learning methods, bidirectional concerns, and stimulation with mutual concessions that lead to higher self-efficacy rates (Ren & Chadee, 2017). Likewise, recent studies, such as those by Goswami and Agrawal (2023), establish a positive relationship between ethical leadership and psychological capital, which specifically increases self-efficacy. In fact, this context of harmony promotes and strengthens shared emotional security that prevents psychological isolation in virtual work environments (Saha et al., 2020) and, in turn, may spur a greater technological self-efficacy oriented towards innovative achievements (Weerawardane & Jayawardana, 2022).

On the other hand, individuals with high levels of self-efficacy show more creativity, flexible tactical criteria, and alternative ways to achieve challenging personal goals when faced with obstacles that initially prevented them (Nunn & Avella, 2015). Therefore, a dominant value system that fosters competitiveness and self-interest is likely to dampen interest in ethical issues but not compromise individual self-efficacy (Swanepoel et al., 2015).

In addition, an egoistic ethical climate inverts the positive relationship between ethical leadership and job self-efficacy. In fact, a benevolent management style maximizes common interests and the homogeneous distribution of personal resources. On the other hand, a climate that only considers its own interests and the organizational interest tends to weaken affective ties, bidirectional support, and identification with ethical principles, regardless of social adjustment. Thus, the amoral emerges and gradually invalidates the role of the ethical leader.

Finally, our findings have practical implications for organizational leaders and human resources professionals. Given the complex relationship between moral reflection and appropriate ethical leadership behavior, we suggest scheduling sessions for ethical leaders at various levels to represent, discuss, and reflect on the moral issues surrounding them. Additionally, our findings indicate that leadership development models with an ethical component, such as high moral awareness, are beneficial. Their implementation can be linked to both increased autonomy and greater self-efficacy. In fact, the ethical leader functions as a valuable organizational resource that boosts employee energy levels, which could help organizations maintain a committed, productive workforce with genuine intentions to stay in their jobs, positively impacting turnover rates.

## Figures and Tables

**Table 1:** Correlation between variables, mean, standard deviation (*n* = 448) CI (95%).

	N	M	SD	EL	EC	SE	JA
Ethical Leadership (EL)	10	49.62	10.130	(0.830)			
Egoistic Ethical Climate (EC)	14	55.60	8.912	0.084 *	(0.590)		
Job Self-efficacy (SE)	6	29.81	3.923	0.314 **	0.104 *	(0.810)	
Job Autonomy (JA)	3	14.91	2.560	0.180 **	0.074	0.368 **	(0.890)

General note: All correlations are significant (* *p* < 0.05, ** *p* < 0.01). Self-prepared.

**Table 2:** Convergent and discriminant validity.

	ALPHA ^1^	CR ^2^	CFC ^3^	AVE ^4^	DV ^5^
Egoistic Ethical Climate	0.77	>1.96	0.730	0.350	0.590
Ethical Leadership	0.92	>1.96	0.830	0.690	0.830
Job Self-efficacy	0.89	>1.96	0.860	0.650	0.810
Job Autonomy	0.87	>1.96	0.850	0.790	0.890

General note: ^1^ Cronbach’s Alpha. ^2^ Critical coefficients. ^3^ Composite reliability. ^4^ Average variance extracted. ^5^ Discriminant Validity. Self-prepared.

**Table 3:** Mediation and moderation ethical leadership vs. self-efficacy.

Effect		Route	β	*p*	t	ES	LLCI	ULCI
Effect EL ^1^ vs. JA ^2^		a1	0.059	0.001	3.636	0.014	0.023	0.078
Gender Covariate vs. JA		---	−0.181	0.522	−0.641	0.229	−0.596	0.303
Seniority Covariate vs. JA		---	0.220	0.017	2.402	0.062	0.027	0.273
Educational level Covariate vs. JA		---	0.170	0.006	2.715	0.072	0.137	0.518
Organizational Position Covariate vs. JA		---	0.130	0.214	1.422	0.044	−0.013	0.478
Effect EL vs. SE ^3^		c1’	0.104	0.001	3.302	0.087	0.117	0.459
Effect JA vs. SE		b1	0.369	0.001	5.900	0.058	0.228	0.456
Effect EEC ^4^ vs. SE		c2’	0.056	0.023	2.276	0.082	0.025	0.346
Effect EL × EEC vs. SE		c3’	−0.061	0.034	−2.129	0.034	−0.013	−0.001
Gender Covariate vs. SE		---	0.152	0.558	0.586	0.277	−0.382	0.706
Seniority Covariate vs. SE		---	0.153	0.032	2.145	0.076	0.014	0.312
Educational level Covariate vs. SE		---	0.190	0.006	2.814	0.052	0.107	0.318
Organizational Position Covariate vs. SE		---	0.110	0.314	1.230	0.024	−0.596	0.256
Conditional Effect EEC (XY)	Low (47)	---	0.135	0.001	6.199	0.022	−0.013	0.177
	Medium (56)	---	0.105	0.001	6.188	0.017	0.072	0.139
	High (64)	---	0.079	0.001	3.741	0.021	0.038	0.121

General note: ^1^ Ethical leadership. ^2^ Job autonomy. ^3^ Self-efficacy. ^4^ Egoistic ethical climate. *f*2 = 0.02 (small), *f*2 = 0.15 (medium), *f*2 = 0.35 (large). (95%) CI (R^2^ = 0.319) (*f*2 = 0.531; High). Self-prepared.

## Data Availability

The original data presented in the study and the used questionnaire are openly available in The Open Science Framework repository at https://osf.io/w2g5b/?view_only=f8b9995262ed469eab5413f302dd83c4 (accessed on 10 January 2025).
